# Corneal Biomechanical Properties in Myopic Eyes Measured by a Dynamic Scheimpflug Analyzer

**DOI:** 10.1155/2015/161869

**Published:** 2015-10-20

**Authors:** Jingyi Wang, Ying Li, Yumei Jin, Xue Yang, Chan Zhao, Qin Long

**Affiliations:** Department of Ophthalmology, Peking Union Medical College Hospital, Chinese Academy of Medical Sciences and Peking Union Medical College, Beijing 100730, China

## Abstract

*Purpose*. To evaluate the corneal biomechanical parameters in myopic and emmetropic eyes using Corneal Visualization Scheimpflug Technology (CorVis ST). *Methods.* 103 myopic and emmetropic eyes of 103 patients were examined. Corneal biomechanical parameters, axial length, and mean keratometry were measured using CorVis ST, IOL Master, and topography, respectively. Corneal biomechanical properties were compared within four groups. Bivariate correlation analysis was used to assess the relationship between corneal biomechanical parameters and ocular characteristics. *Results.* Four of ten corneal biomechanical parameters, namely, deformation amplitude (DA), first- and second-applanation time (A1-time, A2-time), and radius at highest concavity (HC radius), were significantly different within the four groups (*P* < 0.05). In correlation analysis, DA was positively correlated with axial length (*r* = 0.20, *P* = 0.04); A2-time was positively correlated with spherical equivalent (SE) (*r* = 0.24, *P* = 0.02); HC radius was positively correlated with SE (*r* = 0.24, *P* = 0.02) and was negatively correlated with mean keratometry (*r* = −0.20, *P* = 0.046) and axial length (*r* = −0.21, *P* = 0.03). *Conclusions.* The corneal refraction-related biomechanical alterations were associated with ocular characteristics. Highly myopic eyes exhibited longer DA and smaller HC radius than do moderately myopic eyes; the eyes with longer axial length tend to have less corneal stiffness and are easier to deform under stress.

## 1. Introduction

Myopia is a most common ocular disorder and has become a global public health problem. Its worldwide prevalence is over 22% of the current world population and is rising dramatically yearly, reaching 80% in certain Asian countries [1, 2]. Several studies have revealed the correlation between the corneal biomechanical characteristics and myopic degree in children [3] and adult population [4]; nevertheless, the results are still lacking consistence in terms of the biomechanical parameters investigated [3–5]. Although axial length and corneal curvature have been shown to associate with refractive error, the relationship between the two parameters and corneal biomechanical behavior has not been clarified yet [3, 6, 7].

Although it is not an easy task to fulfill a precise evaluation of corneal biomechanical behavior, there are presently two clinical devices, the Ocular Response Analyzer (ORA) (Reichert, Buffalo, New York, USA) and Corneal Visualization Scheimpflug Technology (CorVis ST) (Oculus Optikgeräte GmbH, Wetzlar, Germany), which are commercially available for measuring the corneal biomechanical properties. Corneal hysteresis (CH) and corneal resistance factor (CRF) are the main biomechanical parameters for evaluating the corneal viscoelasticity [8]. Several studies have reported that CH was significantly lower in patients with high myopia, and a relationship between the refractive error and corneal biomechanical properties has also been addressed in adult Spanish and Caucasian population [4, 9]. However, this association failed to show in the study on Singaporean children [6]. CorVis ST is a recently developed noncontact tonometry system integrated with an ultra-high-speed Scheimpflug camera, with 4330 frames per second, which enables recording more biomechanical parameters in response to an air-jet induced deformation. Till now, CorVis ST has been used in the evaluation of healthy eyes [10] and several clinical conditions, such as glaucoma [11] and keratoconus [12, 13], and after refractive procedures [14, 15]. However, the evaluation of corneal biomechanical properties in myopic eyes measured by CorVis ST is limited.

Herein, the aims of this study are twofold: (1) to compare the corneal biomechanical parameters of patients with myopia and normal subjects by CorVis ST and (2) to assess the potential factors which can affect corneal biomechanical behavior, such as refractive error, corneal curvature, and axial length.

## 2. Methods

Unrelated Chinese patients with or without myopia were recruited from the Department of Ophthalmology, Peking Union Medical College Hospital. The study was performed according to the Declaration of Helsinki. Informed consent was obtained from all patients.

All subjects received a complete ophthalmic examination including measurement of best-corrected visual acuity (BCVA), axial length using IOL Master (Carl Zeiss Meditec AG, Jena, Germany), mean keratometry using Topographic Modeling System (TMS-4, TOMEY, Nagoya, Japan), slit-lamp anterior segment biomicroscopy, and fundus examination. Spherical equivalent (SE) was determined by 1 masked and experienced optometrist with noncycloplegic (age ≥ 40 years) or cycloplegic (age < 40 years) refraction using the same Topcon Auto Kerato-Refractometer (KR-8900, Topcon Corporation, Tokyo, Japan). For cycloplegic measurements, 4 drops of Tropicamide Phenylephrine Eye Drops (Santen Pharmaceutical Co., Ltd., Japan) were instilled 10 minutes apart in each eye. The differences of sphere and cylinder value under autorefraction within 3 measurements less than 0.25 D were considered evidence of adequate cycloplegia. Autorefraction measurements were made at least 30 minutes after the last instillation. Subjects were divided into four groups according to their refractive status: Emmetropia group (−0.50 ≤ SE ≤ 0.50), Low myopia group (−0.75 ≤ SE ≤ −3.00 D), Moderate myopia group (−3.25 ≤ SE ≤ −6.00 D), and High myopia group (SE > −6.00 D).

Patients were excluded from the study if they had previous eye surgery, concurrent ocular infectious disease, ocular or systemic diseases (e.g., corneal scars, corneal dystrophy, corneal degradation, keratoconus, glaucoma, uveitis, systemic autoimmune diseases, and diabetes mellitus), or topical eye medication or were corticosteroid users; contact lens wearer and eyes with cylinder greater than 3.0 D were also excluded. Visual acuity was not an exclusion criterion in the current study.

**Figure 1:**
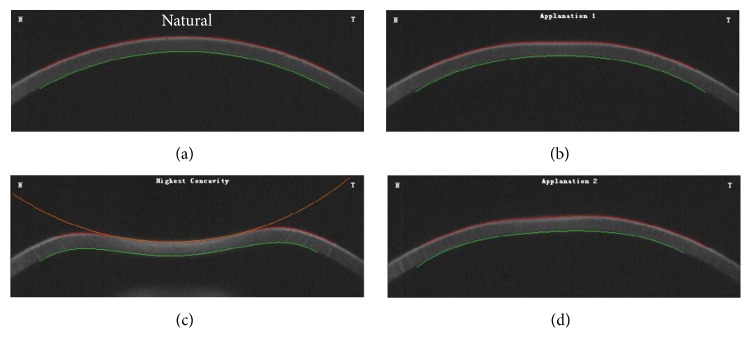
The corneal deformation processes during air puff from CorVis ST. Due to the air puff, the cornea starts with a natural convex shape and undergoes three distinct phases, first applanation, highest concavity, and second applanation, respectively.

Corneal biomechanical parameters were obtained using CorVis ST (Type 72100, Oculus Optikgeräte GmbH, Wetzlar, Germany) by the same investigator in every case to eliminate the possible interobserver variability. A high speed Scheimpflug camera (4330 frames/s) covering 8.0 mm horizontally was applied, which enabled recording 140 Scheimpflug images of the cornea during the deformation in response to a puff of air. Due to the air puff, the cornea underwent three distinct phases, first applanation, highest concavity, and second applanation, respectively (Figure 1). Ten phase-specific parameters generated automatically during the process were as follows: A1-time and A2-time (time from starting until the first and second applanation), A1-length and A2-length (length of the first and second applanation), A1-velocity (A1-V) and A2-velocity (A2-V) (corneal speed during the first- and second-applanation moment), highest concavity-time (HC-time) (time from starting until HC is reached), peak distance (PD) (distance between the two peaks of the cornea at HC), HC radius (central concave curvature at HC), and deformation amplitude (DA) (maximum amplitude at HC) [16]. Intraocular pressure (IOP) and central corneal thickness (CCT) were also obtained during one measurement procedure. CCT was determined by the illustrating snapshot obtained with CorVis ST; IOP was calculated based on the first applanation. To reduce the potential diurnal variations of measured parameters, all the measurements were fulfilled between 8:00 and 11:00 AM.

## 3. Data Analysis

Data were analyzed using IBM SPSS 19.0 for Windows statistical software (SPSS, Chicago, IL) and GraphPad Prism 5 (GraphPad Software, Inc.). Numerical variables were presented as mean ± SD. Kolmogorov-Smirnov (K-S) test was used for testing normal distribution. One-way analysis of variance (ANOVA) and Tukey post hoc tests were used for comparing the parameters of four groups. Pearson's correlation coefficient (*r*) was used to assess the relationship between corneal biomechanical parameters and age, IOP, CCT, SE, axial length, and mean keratometry; Spearman's correlation coefficient (rho) was utilized for determining the relationship between corneal biomechanical parameters and gender. The level of statistical significance was set to *P* < 0.05. Due to the significant correlation for the values between right and left eye, only one randomly selected eye from each subject was analyzed.

## 4. Results

A total of 103 eyes (103 patients) were included in this study. The SE of all included eyes ranged from 0 to −14.00 D. The Emmetropia group (21 eyes of 21 patients) included 17 female and 4 male patients, with a mean age of 34.00 years (range, 21 to 50 years), the Low myopia group (21 eyes of 21 patients) included 13 female and 8 male patients, with a mean age of 30.43 years (range, 21 to 45 years), the Moderate myopia group (28 eyes of 28 patients) included 23 female and 5 male patients, with a mean age of 29.54 years (range, 18 to 44 years), and finally the High myopia group (33 eyes of 33 patients) included 24 female and 9 male patients, with a mean age of 29.29 years (range, 18 to 44 years). Significant differences in SE and axial length were found within the four groups (*F* = 160.1, *P* < 0.001 and *F* = 51.16, *P* < 0.001, resp.). There were no differences within the four groups in terms of age (*F* = 2.21, *P* = 0.09), gender (Kruskal-Wallis test statistic = 3.116, *P* = 0.37), and mean keratometry (*F* = 1.19, *P* = 0.32) (Table 1).

**Figure 2:**
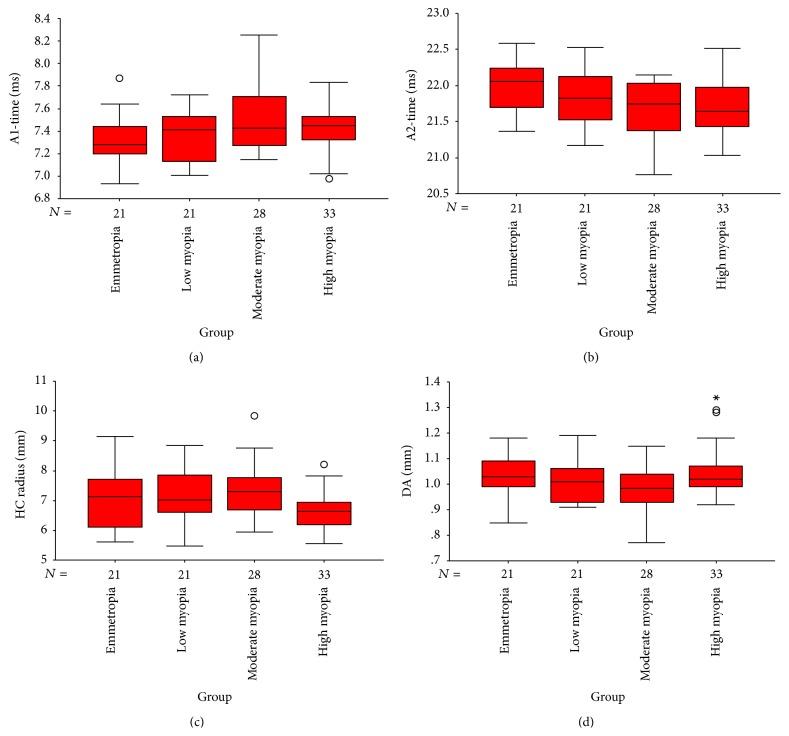
Box plots showing the distribution percentage difference between 4 groups for the A1-time, A2-time, HC radius, and deformation amplitude (DA) levels. The median for each data set is indicated by the center line, and the first and third quartiles are represented by the edges of the area, which is known as the interquartile range (IQR). The 95%/5% confidence intervals are represented by the ends of the lines extending from the IQR. Circles denote outliers with values more than 1.5 IQR from the upper or lower edge of the box.

Four of ten biomechanical parameters, which were deformation amplitude (DA), first- and second-applanation time (A1-time, A2-time), and radius at highest concavity (HC radius), were significantly different within the four groups. In post hoc tests, DA in the High myopia group was significantly higher than in the Moderate myopia group (*q* = 3.86, *P* = 0.008); A1-time in Moderate myopia group was significantly longer than in the Emmetropia group (*q* = 3.99, *P* = 0.006); A2-time of Moderate and High myopia group was significantly longer than that in the Emmetropia group (*q* = 4.03, *P* = 0.005 and *q* = 4.04, *P* = 0.005, resp.); HC radius was significantly smaller in the High myopia group than in the Moderate myopia group (*q* = 6.65, *P* = 0.004). No statistical significance was found within the four groups in terms of A1-length, A2-length, A1-velocity (A1-V), A2-velocity (A2-V), highest concavity-time (HC-time), and peak distance (PD) (all *P* > 0.05). Statistical comparisons for the four groups of the parameters obtained by CorVis ST are shown in Table 2 and Figure 2.

**Figure 3:**
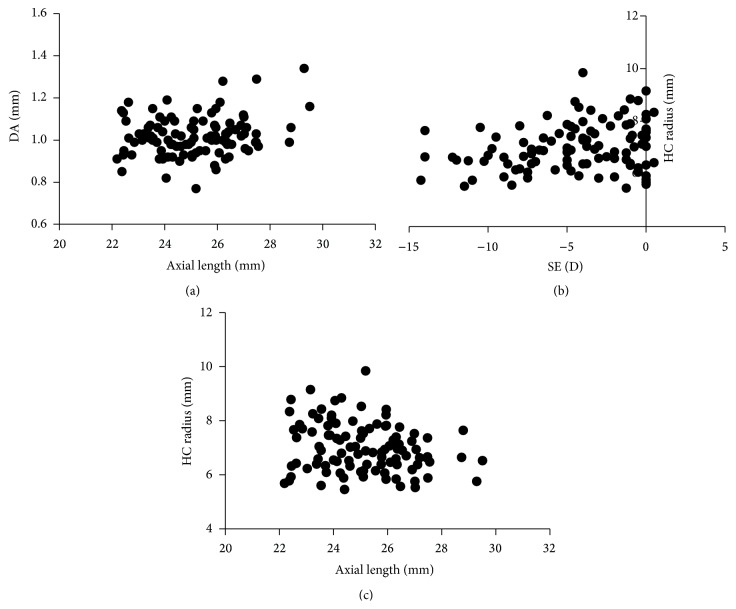
Scatter diagrams of bivariate correlation analysis. (a) Positive correlation between the deformation amplitude (DA) and axial length; (b) positive correlation between the HC radius and spherical equivalent (SE); (c) negative correlation between the HC radius and axial length.

Bivariate correlation analysis was performed to investigate the correlations between the above four significantly different biomechanical parameters of the cornea with potential impact factors, such as age, gender, IOP, CCT, SE, axial length, and mean keratometry. In correlation analysis, DA was positively correlated with age (*r* = 0.33, *P* < 0.001) and axial length (*r* = 0.20, *P* = 0.04) (Figure 3(a)) and negatively correlated with CCT (*r* = −0.35, *P* < 0.001) and IOP (*r* = −0.73, *P* < 0.001); A1-time was positively correlated with CCT (*r* = 0.40, *P* < 0.001) and IOP (*r* = 0.94, *P* < 0.001) and negatively correlated with age (*r* = −0.26, *P* < 0.001); A2-time was positively correlated with age (*r* = 0.31, *P* < 0.001) and SE (*r* = 0.24, *P* = 0.02) and was negatively correlated with IOP (*r* = −0.75, *P* < 0.001); HC radius was positively correlated with SE (*r* = 0.24, *P* = 0.02) (Figure 3(b)), CCT (*r* = 0.27, *P* < 0.001), and IOP (*r* = 0.24, *P* = 0.02) and was negatively correlated with mean keratometry (*r* = −0.20, *P* = 0.046) and axial length (*r* = −0.21, *P* = 0.03) (Figure 3(c)). None of the above four biomechanical parameters was found to be significantly correlated to gender (all *P* > 0.05). The correlation coefficients and *P* values are shown in Table 3.

## 5. Discussion

The cornea is a complex tissue with both viscous and elastic properties; elasticity refers to the deformation of the cornea in response to an external stress, and viscosity refers to the resistance of the cornea in regaining the original shape when the stress is removed [17]. The corneal biomechanical behavior can be affected by a number of factors, such as age, IOP, CCT, hydration, connective tissue composition, and some other factors which are still under investigation [18]. Increased knowledge of corneal biomechanical characteristics in myopic population is of great importance, especially for the preoperative evaluation before refractive surgery. Although several studies have used ORA to identify the corneal biomechanical characteristics of myopic eyes and tried to find the association with certain ocular characteristics, such as refractive error, axial length, and corneal curvature, the results were not consistent with each other. For example, lower CH was significantly associated with longer axial length in 293 Spanish children [3] and 872 Chinese children [5] but not in 271 Singaporean children [6]. And lower CRF was significantly correlated to flatter corneal curvature in a Singapore children study [6] but not in a Chinese children population [7]. To our knowledge, this is the first study to investigate the corneal biomechanics in myopic eyes using CorVis ST, a newly developed dynamic Scheimpflug analyzer, and correlated to ocular characteristics, not only refractive error, but also axial length and corneal curvature.

We found that corneal biomechanical properties, at least some of the parameters achieved by CorVis ST, were significantly altered within different diagnostic groups based on the degree of myopia. Four of ten biomechanical parameters, which were DA, A1-time, A2-time, and HC radius, were significantly different within the four groups in our study. Regarding the factors that affected these parameters, we found that DA was positively correlated to axial length. Since DA is measured from the start of the deformation to the highest concavity, a stiffer cornea would probably be expected to yield lower DA value [12, 19]. For some myopic eyes, the remodeling of the posterior scleral tissue leads to the elongation of the axial length, which in turn contributes to the progression of myopia [20]. Given that the posterior eye is a complex biomechanical structure, the surrounding sclera serves to create a stable biomechanical environment for the ocular tissues [21, 22]. Chang et al. found that lower corneal stiffness was associated with longer axial length [7]; our results consisted with it and suggested that the expansion of the sclera may result in the instability of ocular tissue which consequently reduced the corneal stiffness and caused higher DA value. Since we failed to find the correlation between DA and SE, this hypothesis needs to be confirmed in the future study.

In terms of the HC radius, it showed significant difference within the four groups; in correlation study, the HC radius was positively correlated with SE and negatively correlated with axial length. Since HC radius tends to change in contrast to DA [23], therefore, this result confirmed the finding of DA and suggested that higher myopia and longer axial length result in smaller central concave curvature at the highest concavity.

Increasing evidences show that the corneal biomechanical properties are correlated with IOP and CCT. A lower IOP and thinner central cornea were associated with less stiffness of the cornea and lead to a larger DA and smaller HC radius [24, 25]. It has also been proved in the eyes that underwent corneal refractive surgery, with the weakness of the corneal collagen fibres, which are the main contributors to corneal stiffness, that the cornea tends to have increased indentation during deformation and reduced radius at highest concavity [14, 26]. Our study, as expected, identified that IOP and CCT were negatively correlated with DA and positively correlated with HC radius.

Age is also a potential factor for the corneal biomechanical alterations; age related changes in corneal biomechanical properties have been reported and demonstrated corneal stiffness with age due to the more cross-links of collagen fibrils within the cornea in elderly individuals [27, 28]. Surprisingly, we found a conflicting result which showed a positive instead of theoretically negative correlation between age and DA; however, this is concordant with some other studies, which showed a higher DA value in older individuals [25]. Since most of the elder individuals were excluded from our study because of their systemic diseases, such as coronary heart disease, diabetes mellitus, or the systemic medicine intervention, which could interfere with the interpretation of the study results, the range of age in our study was relatively narrow; further study needs to be conducted to clarify the influence of age on corneal biomechanical properties.

A1-time and A2-time also exhibited significant differences within groups; A1-time and A2-time were the times from starting until the first and second applanation. Both of the parameters are determined not only by the distance from the starting to the first and second applanation, but also by the velocity during the two applanation processes. We found A2-time was positively correlated with SE, which suggested that a higher SE resulted in a longer time to reach the second applanation. This phenomenon needs to be better interpreted in our future studies.

The main limitations of our study are as follows. (1) The sample size in each diagnostic group is relatively small. (2) The age range of the subjects studied was limited. (3) The mean keratometry of the posterior corneal surface was not measured, so the importance of this factor to the corneal biomechanical behavior in myopic population is unknown. (4) Not all the subjects had a general examination and we excluded systemic diseases only by the history; therefore, the potential confounders were not fully excluded. (5) According to the literature, not all of the CorVis ST parameters have ideal repeatability in adults studies, except for IOP, CCT, DA, and A1-time [29, 30], while HC-time, A2-time, and HC radius had low coefficient of variation values [31], so our results still need to be confirmed in the future studies along with the improvement of the equipment design.

In summary, our data showed that there did exist refraction-related biomechanical alterations of the cornea which were associated with ocular characteristics. Highly myopic eyes exhibited longer DA and smaller HC radius than do moderately myopic eyes. The eyes with longer axial length tend to have less corneal stiffness and are easier to deform under stress. This study provided evidences for the application of corneal biomechanical parameters in clinical experience. For example, it has been reported that large DA may induce the underestimation of the IOP measurement [32]; thus, the positive association between DA and myopic degree reminds us of carefully considering IOP value in highly myopic eyes. However, these are our preliminary findings; further large, controlled studies are needed to illustrate highly consistent clinical criteria of corneal biomechanical properties in myopic population, especially for the purpose of ophthalmologic intervention, such as refractive surgery.

Finally, there is one thing which needs to be highlighted, as addressed by Piñero and Alcόn [33]; a lack of enough scientific evidence demonstrating the relationship between biomechanical parameters provided by CorVis ST and the standard mechanical properties limits the ensuring of these parameters in clinical application; therefore, great efforts need to be made to achieve the challenge of developing more accurate devices with which to generate biomechanical parameters closer to the real biomechanical properties of the cornea; thus clear-cut conclusions may be drawn.

## Figures and Tables

**Table 1:** The demographic data of the study population.

Parameter	Emmetropia (*n* = 21)	Low myopia (*n* = 21)	Moderate myopia (*n* = 28)	High myopia (*n* = 33)	*P* value
Age (years)	34.00 ± 7.85	30.43 ± 6.43	29.54 ± 6.77	29.29 ± 6.97	0.09^a^
Sex (M/F)	17/4	13/8	23/5	24/9	0.37^b^
SE (D)	−0.07 ± 0.28	−1.71 ± 0.78	−4.41 ± 0.73	−8.98 ± 2.66	<0.001^a^
AL (mm)	23.21 ± 0.94	24.34 ± 1.08	25.28 ± 0.82	26.69 ± 1.27	<0.001^a^
MK (D)	43.85 ± 0.99	43.44 ± 1.23	43.40 ± 1.36	43.84 ± 1.02	0.32^a^

M: male; F: female; D: diopters; SE: spherical equivalent; AL: axial length; MK: mean keratometry.

^a^One-way analysis of variance.

^b^ *χ*-test.

Significant differences in SE and AL were present among the four groups (post hoc test, *P* < 0.05).

**Table 2:** All the parameters obtained by CorVis ST for the 4 groups, mean ± SD.

Parameters	Emmetropia (*n* = 21)	Low myopia (*n* = 21)	Moderate myopia (*n* = 28)	High myopia (*n* = 33)	*P* value
A1-time (ms)	7.30 ± 0.23	7.37 ± 0.22	7.50 ± 0.28^#^	7.42 ± 0.20	0.04
A2-time (ms)	21.88 ± 0.33	21.84 ± 0.37	21.68 ± 0.38^#^	21.69 ± 0.39^†^	0.02
A1-length (mm)	1.76 ± 0.05	1.77 ± 0.03	1.76 ± 0.08	1.78 ± 0.04	0.68
A2-length (mm)	1.67 ± 0.34	1.75 ± 0.21	1.70 ± 0.24	1.74 ± 0.25	0.71
A1-velocity (m/s)	0.15 ± 0.01	0.14 ± 0.01	0.14 ± 0.02	0.15 ± 0.01	0.10
A2-velocity (m/s)	−0.32 ± 0.05	−0.31 ± 0.08	−0.30 ± 0.05	−0.33 ± 0.07	0.37
HC-time (ms)	17.18 ± 0.47	17.06 ± 0.57	17.07 ± 1.10	17.87 ± 0.49	0.46
PD (mm)	3.80 ± 1.17	3.78 ± 1.17	4.15 ± 1.13	3.69 ± 1.22	0.47
HC radius (mm)	7.09 ± 1.08	7.12 ± 0.91	7.32 ± 0.89	6.65 ± 0.66^*∗*^	0.03
DA (mm)	1.03 ± 0.08	1.01 ± 0.09	0.98 ± 0.09	1.05 ± 0.10^*∗*^	0.048
IOP (mmHg)	13.69 ± 2.04	13.93 ± 2.11	15.03 ± 2.67	14.33 ± 1.98	0.17
CCT (*μ*m)	537.3 ± 34.6	546.4 ± 30.0	541.7 ± 21.7	533.6 ± 32.2	0.49

A1- and A2-time: time reaching the first and second applanation; A1- and A2-length: length of the first and second applanation; A1- and A2-velocity: velocity at the first- and second-applanation moment; HC-time: highest concavity- (HC-) time; PD: peak distance; HC radius: radius at HC; DA: deformation amplitude; IOP: intraocular pressure; CCT: central corneal thickness.

^*∗*^ *P* < 0.05 versus Moderate myopia group.

^#^ *P* < 0.05 versus Emmetropia group.

^†^ *P* < 0.05 versus Emmetropia group.

**Table 3:** Factors associated with corneal parameters with bivariate correlation analysis.

Parameters	A1-time (*n* = 103)		A2-time (*n* = 103)		HC radius (*n* = 103)		DA (*n* = 103)	
	Coeff.	*P*	Coeff.	*P*	Coeff.	*P*	Coeff.	*P*
Age	−0.26	0.01	0.31	0.002	−0.05	0.66	0.33	<0.001
Sex	0.01	0.96	0.04	0.67	0.05	0.61	0.05	0.64
SE (D)	−0.15	0.13	0.24	0.02	0.24	0.02	−0.13	0.18
AL (mm)	0.07	0.46	−0.15	0.15	−0.21	0.03	0.20	0.04
MK (D)	0.07	0.49	−0.18	0.08	−0.20	0.046	−0.003	0.97
IOP (mmHg)	0.94	<0.001	−0.75	<0.001	0.24	0.02	−0.73	<0.001
CCT (*μ*m)	0.40	<0.001	−0.17	0.09	0.27	0.01	−0.35	<0.001

D: diopters; SE: spherical equivalent; AL: axial length; MK: mean keratometry; A1- and A2-time: time reaching the first and second applanation; HC radius: radius at highest concavity; DA: deformation amplitude; IOP: intraocular pressure; CCT: central corneal thickness; Coeff.: the correlation coefficient.
